# Morphine Plus Placebo vs Morphine Plus Acetaminophen for Acute Pain in the Emergency Department: A Randomized Clinical Trial

**DOI:** 10.1001/jamanetworkopen.2025.60250

**Published:** 2026-02-24

**Authors:** Guillaume Cattin, Damien Viglino, Julien Segard, Christelle Volteau, Anthony Chauvin, Michel Galinski, Olivier Maigre, Alix Delamare-Fauvel, Marion Le Pottier, Valerie Debierre, Tahar Chouihed, Céline Longo, Joël Jenvrin, Yonathan Freund, Emmanuel Montassier

**Affiliations:** 1 Service des Urgences, Nantes Université, Centre Hospitalier Universitaire Nantes, Nantes, France; 2 Emergency Department and HP2 Laboratory INSERM U1800, Centre Hospitalier Universitaire Grenoble Alpes, Grenoble, France; 3 Service des Urgences, Centre Hospitalier Saint Nazaire, Nantes, France; 4 Centre Hospitalier Universitaire Nantes, Research and Innovation Direction, Methodology and Biostatistics Platform, Nantes University, Nantes, France; 5 Assistance Publique-Hôpitaux de Paris, Hôpital Lariboisière, Service d’Accueil des Urgences et Service Mobile d’Urgence et de Réanimation, Paris, France; 6 Service d’Aide Médicale Urgente, Hôpital Pellegrin, Centre Hospitalier Universitaire de Bordeaux, Bordeaux Cedex, France; 7 INSERM 1219, Bordeaux Population Health Research Centre, AHeaD Team, Université de Bordeaux, Bordeaux, France; 8 Service des Urgences, Centre Hospitalier Lorient, Nantes, France; 9 Emergency Department, Rouen University Hospital, Rouen, France; 10 Département de Médecine d’Urgences, Centre Hospitalier Universitaire Angers, Angers, France; 11 Service d’Urgences, Centre Hospitalier La Roche sur Yon, La Roche sur Yon, France; 12 Service d’Urgences, Centre Hospitalier Universitaire de Nancy, Université de Lorraine, Nancy, France; 13 Emergency Department, Hôpital Pitie-Salpêtrière, Sorbonne Université, Paris, France; 14 INSERM, Centre Hospitalier Universitaire Nantes, Center for Research in Transplantation and Translational Immunology, Unité Mixte de Recherche 1064, Nantes Université, Nantes, France

## Abstract

**Importance:**

Intravenous (IV) acetaminophen is widely used in multimodal analgesia with opioids for the initial management of acute pain in emergency departments (EDs), but its additive benefit when combined with titrated morphine is uncertain.

**Objective:**

To determine whether titrated IV morphine plus placebo is noninferior to titrated IV morphine plus acetaminophen for pain relief in the first hour in adults seen in the ED with acute traumatic or nontraumatic pain.

**Design, Setting, and Participants:**

This multicenter, double-blind, randomized, placebo-controlled noninferiority clinical trial was conducted in 11 French EDs from December 3, 2019, to December 31, 2024, with 60 minutes of follow-up. Adults (aged ≥18 years) with severe acute pain (numeric rating scale [NRS] score ≥5 on a scale from 0 [no pain] to 10 [worst pain]) of traumatic or nontraumatic origin were eligible. Prespecified analyses were planned for traumatic and nontraumatic pain.

**Interventions:**

All patients received weight-based titrated IV morphine. In the control group, patients also received 1 g IV acetaminophen; in the intervention group, patients received matching placebo.

**Main Outcomes and Measures:**

The primary outcome was the mean change in NRS pain score from baseline to 30 minutes. The noninferiority margin was 1 point. Analyses were conducted in modified intention-to-treat (mITT) and per-protocol (PP) populations.

**Results:**

Among 430 randomized patients (median age, 42 years [IQR, 29-57 years]; 220 men [51.2%]), 424 were included in the mITT analysis (213 in the morphine plus placebo and 211 in the morphine plus acetaminophen group; 181 [42.7%] had traumatic pain and 243 [57.3%] had nontraumatic pain) and 393 were included in the PP analysis (197 morphine plus placebo and 196 morphine plus acetaminophen; 169 [43.0%] had traumatic pain and 224 [57.0%] had nontraumatic pain). In the PP analysis, the between-group difference in mean pain score reduction was 0.32 points (95% CI, −0.29 to 0.94 points) for traumatic pain and 0.80 points (95% CI, 0.19-1.41 points) for nontraumatic pain. In the mITT analysis, the corresponding differences were 0.36 points (95% CI, −0.28 to 1.01 points) and 0.76 points (95% CI, 0.11-1.41 points). In both strata, pain reduction favored the acetaminophen group, but the differences did not meet the prespecified noninferiority margin of 1 point.

**Conclusions and Relevance:**

In this randomized clinical trial of adults seen in the ED with acute pain, morphine plus placebo did not meet the criterion for noninferiority compared with morphine plus acetaminophen for pain relief during the initial management. The findings suggest acetaminophen may have potential benefit as an adjunct to morphine in individualized treatment approaches for acute pain in the ED.

**Trial Registration:**

ClinicalTrials.gov Identifier: NCT04148495

## Key Points

**Question:**

Among adults seen in the emergency department with acute pain, does morphine plus placebo provide noninferior initial pain relief compared with morphine plus acetaminophen?

**Findings:**

In this randomized clinical trial involving 430 patients, differences in pain scores after treatment with intravenous morphine plus placebo vs intravenous morphine plus acetaminophen did not meet the prespecified noninferiority margin of 1 point in patients with either traumatic or nontraumatic pain.

**Meaning:**

In this study, morphine plus placebo did not demonstrate noninferiority to morphine plus acetaminophen for initial pain relief.

## Teaser

This randomized clinical trial investigates whether intravenous morphine plus placebo is noninferior to morphine plus acetaminophen for initial management of acute pain in adult patients in the emergency department.

## Introduction

Pain is a leading cause of emergency department (ED) visits, and opioids remain the mainstay of treatment for moderate-to-severe acute pain.^1,2,3^ Concerns about opioid-related adverse effects and potential misuse have increased interest in multimodal analgesia to reduce opioid exposure.^4,5,6^ Intravenous (IV) acetaminophen is frequently used as an adjunct in ED analgesic regimens, although its additive benefit when combined with titrated IV morphine remains uncertain.^7,8,9^

Prior randomized trials in the ED have generally not shown clinically meaningful improvements in pain scores or opioid-sparing effects with IV acetaminophen, but many studies were limited by inadequate power, heterogenous pain etiologies, fixed dosing strategies, and variability in opioid regimens.^7,8,9,10,11,12,13^ Despite this uncertainty, the combination of IV acetaminophen and opioids remains widespread in French ED practice. Accordingly, we used a noninferiority trial design to evaluate whether titrated IV morphine alone could provide analgesia that is not meaningfully worse than morphine plus acetaminophen during the first hour of ED management. We hypothesized that morphine plus placebo would be noninferior to morphine plus acetaminophen in adults with acute traumatic and nontraumatic pain.

## Methods

### Study Design

This study was a prospective, multicenter, double-blind, placebo-controlled noninferiority randomized clinical trial comparing titrated IV morphine plus placebo with titrated IV morphine plus acetaminophen for pain relief during the first hour in adults seen in the ED with traumatic or nontraumatic acute pain. The trial was conducted in 11 French EDs from December 3, 2019, to December 31, 2024. Participants and physicians were both blinded to treatment allocation. The study protocol was reviewed and approved by the Comité de Protection des Personnes CPP OUEST I ethics committee on October 10, 2019. Written informed consent was obtained from all participants prior to enrollment. The protocol is provided in Supplement 1, with additional methods in the eMethods in Supplement 2. The trial was conducted in accordance with International Council for Harmonisation good clinical practice, with adherence to the ethical principles of the Declaration of Helsinki (1964 and subsequent amendments),^14^ and followed the Consolidated Standards of Reporting Trials (CONSORT) reporting guideline.

### Patient Population

Adults (aged ≥18 years) were eligible if they had acute pain of less than 24 hours’ duration with a numeric rating scale (NRS) score of 5 or higher (0 = no pain; 10 = worst pain), were able to communicate, and could rate pain.^15,16,17,18^ Exclusion criteria were unstable vital signs, Glasgow Coma Scale score less than 15 (score range, 3 [lowest consciousness] to 15 [highest consciousness]), pregnancy, body weight less than 50 kg, need for immediate fracture treatment or joint reduction, acute cardiopulmonary emergencies (eg, pulmonary edema, respiratory failure, or acute coronary syndrome), acute intoxication, analgesic use within 8 hours, inability to obtain venous access, hypersensitivity to study drugs, kidney or hepatic insufficiency, chronic pain treatment, or use of buprenorphine, nalbuphine, or pentazocine.^19^

### Randomization and Allocation

Patients were randomized in a 1:1 ratio to receive either titrated IV morphine plus placebo or titrated IV morphine combined with acetaminophen. Randomization was based on a computer-generated sequence established prior to study initiation. The process was centralized and conducted through a secure, web-based platform managed by an independent biostatistician who was not involved in patient enrollment. Allocation was balanced using permuted blocks of varying length (block sizes of 4 and 6) and stratified by pain type (traumatic vs nontraumatic).

### Study Intervention

In the control group, patients received a single dose of IV acetaminophen in addition to titrated IV morphine. In the intervention group, patients received a single dose of a matching placebo in addition to titrated IV morphine. Randomization kits contained either 1 g of acetaminophen in a 100-mL ready-to-use IV bag or 100 mL of 0.9% sodium chloride (placebo), administered in a double-blind fashion concurrently with titrated IV morphine. In both groups, an initial IV bolus of morphine at 0.10 mg/kg (maximum dose, 10 mg) was administered over 2 minutes.^20^ Subsequent boluses of morphine at 0.05 mg/kg (maximum, 5 mg per bolus) were titrated every 10 minutes until 1 of the following criteria was met: adequate pain relief (defined as an NRS pain score ≤3^11^), occurrence of a serious adverse event (eg, profound hypotension, loss of consciousness, or respiratory depression requiring ventilatory support), or administration of a cumulative morphine dose of 20 mg within the first 30 minutes. Emergency physicians adjusted dosing based on their clinical judgment, taking into account the patient’s age and body habitus.^4^ If pain relief was not achieved despite multiple morphine doses, rescue analgesia was administered at the discretion of the treating physician, including choice and dosage of the additional agent. For patients whose peripheral oxygen saturation dropped below 94% during drug administration, supplemental oxygen was delivered via nasal cannula at a flow rate of 2 L/min, adjusted as needed according to follow-up oxygen saturation measurements.

### Outcomes

Pain intensity was assessed using an 11-point NRS in which a score of 0 indicates no pain and a score of 10 indicates the worst possible pain.^4,21,22,23^ The primary outcome was the mean change in verbal NRS pain scores from baseline to 30 minutes after study drug administration.

Secondary outcomes included mean NRS change at 10, 20, 45, and 60 minutes; cumulative morphine dose within 30 minutes (mg/kg); successful analgesia at 30 minutes (NRS ≤3); and rescue analgesia at 30 minutes. Additional outcomes were changes in vital signs at 10, 20, 45, and 60 minutes and adverse events (nausea or vomiting, oxygen saturation <94%, systolic blood pressure <90 mm Hg, dizziness, Glasgow Coma Scale score ≤13, rash, or pruritus). Investigators actively monitored adverse events during the 60-minute observation period and recorded all events in the electronic case report form. Patients were also followed up for 24 hours after the last morphine dose to capture delayed adverse events.

### Sample Size

The hypotheses for sample size calculations were primarily based on the results of the most recent and methodologically rigorous, to our knowledge, randomized clinical trial evaluating analgesic efficacy for acute pain in the emergency department.^4^ In that study, which compared 4 oral analgesics, the lower bound of the 95% CI for the between-group difference in mean change in NRS pain scores from baseline to 1 hour was –1. Additional support for this margin comes from prior investigations of the minimally clinically important difference in pain measurement. Kelly^24^ found that the minimum clinically significant difference in visual analog scale pain scores could be as low as 0.9 cm, while Todd^25^ reported that a difference of 1 unit represents a clinically perceptible change in pain intensity. In the present trial, we deliberately selected a conservative margin of 1 point, representing the smallest difference that would still be considered clinically meaningful. This choice was made to ensure a robust and clinically cautious interpretation of noninferiority, minimizing the risk of incorrectly declaring the treatments noninferior when a clinically meaningful difference exists.

Using the parameters of 2-sided significance level (α) of .05 divided by 2, 90% power, noninferiority margin of 1, and within-group SD of 2.6, we estimated that 143 patients per treatment arm would be required within each pain stratum (traumatic and nontraumatic), yielding a total sample size of 572 participants.^19^ However, the COVID-19 pandemic caused prolonged interruptions of recruitment and substantial reductions in eligible ED presentations across participating centers. On October 15, 2022, after 242 participants had been enrolled and prior to any unblinding, the powering strategy was revised to 80% following consultation with the sponsor and biostatistics team to ensure feasibility. Eighty percent power is widely accepted for randomized and noninferiority clinical trials.^26^ The revised calculation yielded a required total sample size of 428 participants (107 per treatment arm within each pain stratum).

### Statistical Analysis

Characteristics at baseline were described by their frequency (percentage) for categorical variables and by means (SDs) or medians (IQRs) for quantitative variables. A linear model was used to model the change in NRS pain scores from baseline to 30 minutes. The model was adjusted on baseline NRS value. The primary end point was analyzed by computing a 95% CI for the mean difference in NRS change, comparing IV morphine plus placebo with IV morphine combined with acetaminophen. Noninferiority was established if the higher bound of this 95% CI was inferior to 1. Because this was a noninferiority trial, the primary analysis included both the per-protocol (PP) population (all randomized and treated patients without major protocol deviations, including nonadherence to the assigned intervention, use of prohibited concomitant medications, or missing primary outcome data) and the modified intention-to-treat (mITT) population (all randomized patients except those who withdrew consent, were under guardianship, or were younger than 18 years), in accordance with the CONSORT extension for noninferiority and equivalence trials.^27,28^ Noninferiority was to be concluded only if both the PP and mITT analyses supported it. To address missing pain assessments at 30 minutes, a worst-case imputation strategy was applied. For these patients, the smallest observed NRS change from baseline to 30 minutes among those with the same pain category and treatment group was imputed.

The secondary efficacy end points were tested for noninferiority in the PP and the mITT populations. The same noninferiority margin of 1 point on the NRS scale was applied. The other secondary end points were tested for superiority in the mITT population. Qualitative secondary end points were analyzed with the χ^2^ test or the Fisher exact test when necessary. Proportion differences and the corresponding 95% CIs were estimated. For quantitative secondary end points, the 2-tailed *t* test or Mann-Whitney *U* test was used according to gaussian or nongaussian statistical distribution. Linear mixed models were used to compare repeated quantitative variables over time between treatment groups. The significance threshold was 2-sided *P* < .05 without adjustment for multiplicity. Analyses used SAS software, version 9.4 (SAS Institute Inc). The detailed statistical analysis plan is available in Supplement 1.

## Results

### Baseline Characteristics

**Figure 1.**
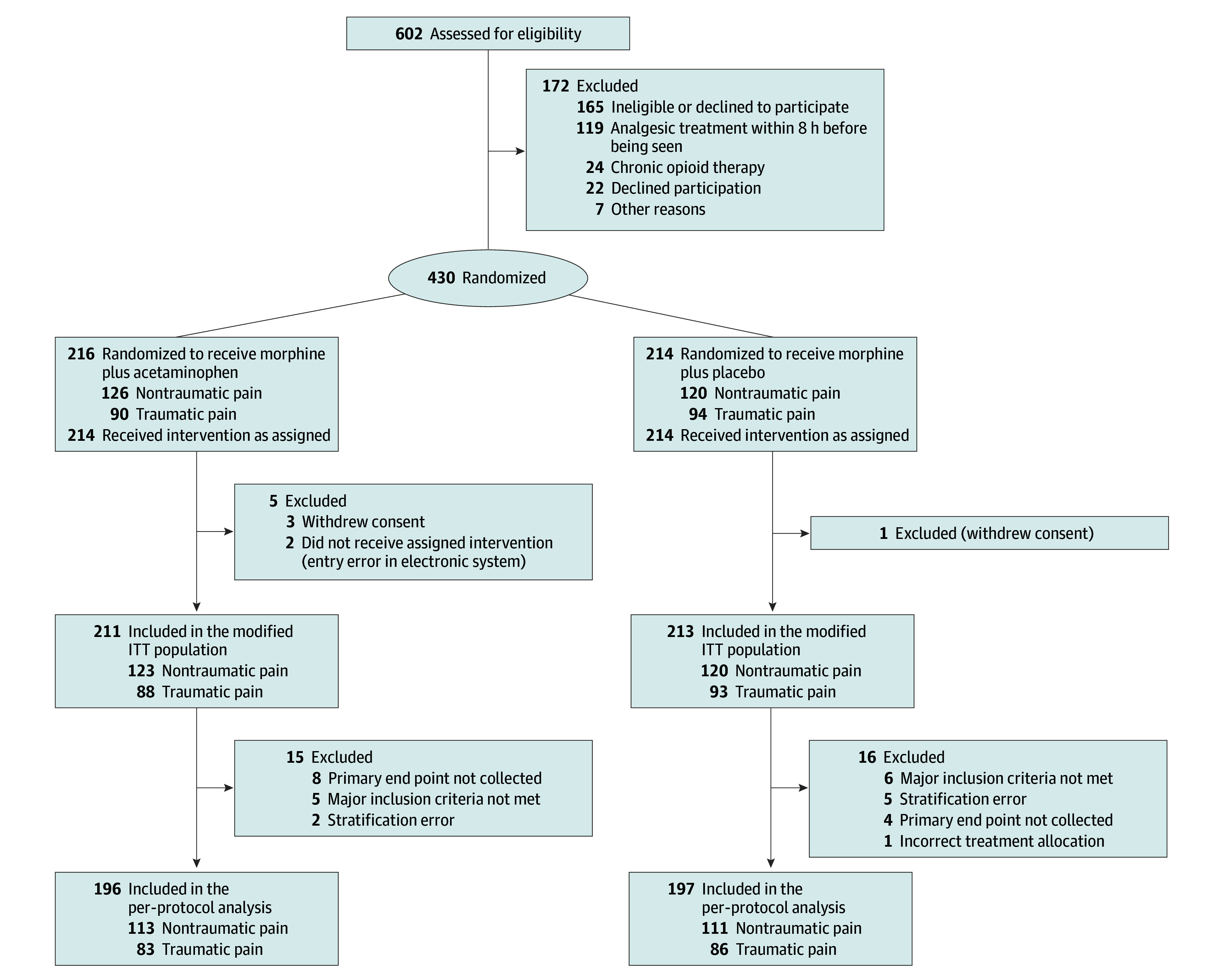
Study Flow Diagram ITT indicates intention to treat.

A total of 430 patients were enrolled (median age, 42 years [IQR, 29-57 years]); 220 (51.2%) were men and 210 (48.8%) were women. Overall, 214 patients were randomized to morphine plus placebo and 216 to morphine plus acetaminophen (Figure 1). The mITT population included 424 patients, 213 in the morphine plus placebo and 211 in the morphine plus acetaminophen group; 181 (42.7%) had traumatic pain and 243 (57.3%) had nontraumatic pain. The PP population (n = 393) included 197 patients in the morphine plus placebo and 196 in the morphine plus acetaminophen group; 169 (43.0%) had traumatic and 224 (57.0%) had nontraumatic pain (Figure 1). Baseline characteristics are shown in Table 1, and enrollment by center is provided in eTable 2 in Supplement 2.

**Table 1.** Demographic Data and Clinical Characteristics of Patients

Characteristic	Patient group^a^		
	All (N = 424)	Morphine plus acetaminophen (n = 211)	Morphine plus placebo (n = 213)
Sex^b^			
Men	212 (50)	96 (45)	116 (54)
Women	210 (50)	113 (54)	97 (46)
Age, median (IQR) [range], y^b^	42 (29-57) [18-94]	40 (29-55) [18-94]	43 (30-58) [18-88]
Estimated BMI, median (IQR)^b^	24.5 (21.9-28.0)	24.3 (22.1-28.1)	24.6 (21.5-28.0)
Medical history			
Hypertension	68 (16)	32 (15)	36 (17)
Cancer	14 (3)	2 (1)	12 (6)
Neurologic disorder	7 (2)	4 (2)	3 (1)
Diabetes	19 (4)	6 (3)	13 (6)
Coronary heart disease	5 (1)	2 (1)	3 (1)
COPD	1 (1)	1 (1)	1 (1)
Chronic kidney failure	4 (1)	2 (1)	2 (1)
Liver disease	1 (1)	0	1 (1)
Characteristics at enrollment, median (IQR)^c^			
Heart rate, beats/min	77 (69-88)	78 (71-87)	76 (68-89)
Respiratory rate, breaths/min	18 (16-20)	18 (16-20)	20 (16-21)
Arterial blood pressure, mm Hg			
Systolic	131 (119-149)	131 (117-150)	131 (120-148)
Diastolic	79 (70-88)	80 (69-87)	79 (70-88)
Peripheral oxygen saturation, %	98 (97-100)	98 (97-99)	98 (97-100)
GCS score^d^	15 (15-15)	15 (15-15)	15 (15-15)
Nature of pain^c^			
Nontraumatic	243 (57)	123 (58)	120 (56)
Chest	13 (5)	5 (4)	8 (6)
Abdominal	181 (74)	91 (74)	90 (75)
Headache	18 (7)	10 (7)	8 (6)
Back	26 (10)	14 (11)	12 (10)
Other^e^	9 (4)	5 (4)	4 (3)
Traumatic	181 (43)	88 (42)	93 (44)
Head and neck	27 (15)	12 (14)	15 (16)
Maxillofacial	14 (7)	10 (11)	4 (4)
Chest	28 (15)	15 (17)	13 (14)
Abdominal	18 (10)	12 (13)	6 (6)
Extremity	158 (88)	78 (89)	80 (86)
Skin or soft tissue	27 (15)	15 (17)	12 (12)
Injury severity score, median (IQR) [range]^f^	3 (2-3) [0-8]	3 (2-3) [0-7]	3 (2-3) [1-8]
Initial pain score, median (IQR) [range]^b^	8 (7-9) [5-10]	8 (7-10) [5-10]	8 (7-9) [5-10]

Abbreviations: BMI, body mass index (calculated as weight in kilograms divided by height in meters squared); COPD, chronic obstructive pulmonary disease; GCS, Glasgow Coma Scale.

^a^ Unless otherwise indicated, data are expressed as the number (percentage) of patients. Percentages have been rounded and may not sum to 100.

^b^ Data were missing for 2 patients.

^c^ Data were missing for 4 patients.

^d^ Scores range from 3 (lowest consciousness) to 15 (highest consciousness).

^e^ Included sacrococcygeal abscess, anal pain, facial rash, toothache, throat and ear pain, lower limb pain, neuralgia, sore throat, and testicular pain.

^f^ Data were missing for 1 patient.

### Primary Outcome

**Figure 2.**
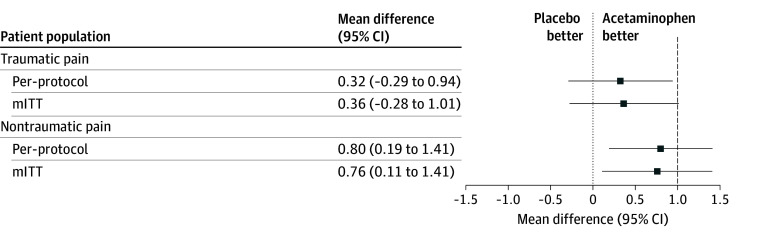
Forest Plot of Mean Differences in Pain Reduction Scores at 30 Minutes Between the Acetaminophen and Placebo Groups Dashed vertical line represents the predefined noninferiority margin of 1. mITT indicates modified intention to treat.

Among patients with traumatic acute pain in the PP population, the mean reduction in pain score from baseline to 30 minutes was −4.50 points (95% CI, −4.93 to −4.08 points) with placebo and −4.83 points (95% CI, −5.26 to −4.39 points) with acetaminophen. The between-group difference was 0.32 points (95% CI, −0.29 to 0.94 points), which fell within the predefined noninferiority margin of 1 point (Figure 2). In the mITT population, there was a between-group difference of 0.36 points (95% CI, −0.28 to 1.01 points). Because the upper bound of the 95% CI exceeded 1 point, and both analyses were required to meet noninferiority criteria, the mITT analysis did not support noninferiority for traumatic pain.

Among patients with nontraumatic acute pain in the PP population, the mean reduction in pain score was −4.77 points (95% CI, −5.20 to −4.33 points) with placebo and −5.57 points (95% CI, −6.00 to −5.14 points) with acetaminophen, resulting in a between-group difference of 0.80 points (95% CI, 0.19-1.41 points). This difference exceeded the noninferiority margin (Figure 2). Findings were consistent in the mITT analysis, with a between-group difference of 0.76 points (95% CI, 0.11-1.41 points), which also exceeded the noninferiority margin.

### Secondary Outcomes

Pain intensity decreased over time in both groups (eFigure in Supplement 2). At 60 minutes, in the subgroup with traumatic pain, the mean reduction was −5.12 points (95% CI, −5.53 to −4.72 points) with placebo vs −5.52 points (95% CI, −5.94 to −5.11 points) with acetaminophen (PP difference, 0.40 [95% CI, −0.18 to 0.98] points; mITT difference, 0.44 [95% CI, −0.14 to 1.01] points); the upper bound of the 95% CI was within the prespecified noninferiority margin of 1 point in the PP analysis but not in the mITT analysis. In the subgroup with nontraumatic pain, the mean reduction was −5.84 points (95% CI, −6.23 to −5.85 points) vs −6.07 points (95% CI, −6.46 to −5.69 points) (PP difference, 0.23 [95% CI, −0.32 to 0.78] points; mITT difference, 0.24 [95% CI, −0.32 to 0.79] points), with the upper bounds of both 95% CIs within the prespecified margin. The median weight-adjusted morphine dose at 30 minutes and the proportion of patients with successful analgesia (NRS ≤3) were similar between groups (Table 2). Rescue analgesia at 30 minutes was more frequent with placebo in the subgroup with nontraumatic pain, with no difference in the subgroup with traumatic pain. No clinically meaningful differences in vital signs were observed (eTables 3 and 4 in Supplement 2).

**Table 2.** Secondary End Points by Study Group

End point	Patient group^a^		Adjusted mean difference (95% CI)^b^	*P* value
	Morphine plus acetaminophen	Morphine plus placebo		
Total amount of morphine at 30 min, mean (SD)				
Milligrams^c^				
Nontraumatic pain	11.0 (4.4) [n = 120]	12.1 (4.3) [n = 116]	−1.1 (−2.2 to 0.0)	.10
Traumatic pain	12.0 (4.6) [n = 86]	11.3 (4.7) [n = 91]	0.7 (−0.7 to 2.1)	.32
Milligrams per kilogram^d^				
Nontraumatic pain	0.16 (0.1) [n = 120]	0.17 (0.1) [n = 116]	−0.01 (−0.03 to 0.00)	.07
Traumatic pain	0.21 (0.1) [n = 86]	0.20 (0.1) [n = 91]	0.01 (0.00 to 0.03)	.16
Successful analgesia at 30 min, No./total No. (%)^e^				
Nontraumatic pain	81/117 (69)	71/115 (62)	10.0 (−4.7 to 19.7)	.23
Traumatic pain	57/85 (67)	59/90 (66)	−2.0 (−15.5 to 12.5)	.83
Rescue analgesia at 30 min, No./total No. (%)^f^				
Nontraumatic pain	3/120 (2)	15/117 (13)	−12.0 (−17.0 to −3.7)	.01
Traumatic pain	1/86 (1)	2/90 (2)	−1.0 (−4.8 to 2.7)	.99

^a^ Percentages have been rounded and may not sum to 100.

^b^ Positive values favor the morphine plus acetaminophen group; negative values favor the morphine plus placebo group.

^c^ Data were missing for 7 patients.

^d^ Data were missing for 5 patients.

^e^ Data were missing for 4 patients.

^f^ Data were missing for 1 patient.

### Adverse Events Analysis

At 30 minutes, the overall incidence of adverse effects was low and generally similar between groups (Table 3). Among patients with traumatic pain, the most common adverse event was nausea, reported in 8.6% (95% CI, 2.9%-14.3%) of patients receiving titrated IV morphine plus placebo and 10.3% (95% CI, 4.0%-16.7%) of those receiving titrated IV morphine plus acetaminophen (adjusted mean difference, 1.7 percentage points; 95% CI, −0.1 to 0.1 percentage points). In the subgroup with nontraumatic pain, nausea was reported in 28.0% (95% CI, 18.8%-37.1%) of patients in the titrated IV morphine plus placebo group and 29.5% (95% CI, 20.0%-39.1%) in the titrated IV morphine plus acetaminophen group (adjusted mean difference, 1.6 percentage points; 95% CI, −11.6 to 14.8 percentage points).

**Table 3.** Frequency of Adverse Effects Observed by Study Group

Adverse effect	Patient group				Adjusted mean difference (95% CI), %
	Morphine plus acetaminophen (n = 123)		Morphine plus placebo (n = 120)		
	Patients, No.	Risk (95% CI), %	Patients, No.	Risk (95% CI), %	
**Nontraumatic pain**					
Nausea	26	29.5 (20.0 to 39.1)	26	28.0 (18.8 to 37.1)	1.6 (−11.6 to 14.8)
Vomiting	10	11.4 (4.7 to 18.0)	9	9.7 (3.7 to 15.7)	1.7 (−7.3 to 10.6)
Respiratory depression	5	5.7 (0.8 to 10.5)	4	4.3 (0.2 to 8.4)	1.4 (−5.0 to 7.7)
Hypotension	4	4.5 (0.2 to 8.9)	0	0.0 (0.0 to 0.0)	4.5 (0.2 to 8.9)
Dizziness	10	11.4 (4.7 to 18.0)	14	15.1 (7.8 to 22.3)	−3.7 (−13.5 to 6.1)
Decreased consciousness (GCS score ≤13)^a^	4	4.5 (0.2 to 8.9)	2	2.2 (−0.8 to 5.1)	2.4 (−2.9 to 7.7)
Rash or pruritus	1	1.1 (−1.1 to 3.4)	2	2.2 (−0.8 to 5.1)	−1.0 (−4.7 to 2.7)
**Traumatic pain**					
Nausea	9	10.3 (4.0 to 16.7)	8	8.6 (2.9 to 14.3)	1.7 (−0.07 to 0.10)
Vomiting	2	2.3 (0.0 to 5.4)	4	4.3 (0.1 to 8.4)	−2.0 (−7.2 to 3.2)
Respiratory depression	1	1.1 (−1.1 to 3.4)	1	1.1 (−1.1 to 3.2)	0.1 (−3.0 to 3.1)
Hypotension	0	0.0 (0.0 to 0.0)	0	0.0 (0.0 to 0.0)	0.0 (0.0 to 0.0)
Dizziness	3	3.4 (0.0 to 7.3)	3	3.2 (0.0 to 6.8)	0.2 (−5.0 to 5.5)
Decreased consciousness (GCS score ≤13)^a^	7	8.0 (2.3 to 13.6)	4	4.3 (0.2 to 8.4)	3.7 (−3.3 to 10.7)
Rash or pruritus	2	2.3 (0.0 to 5.5)	1	1.1 (0.0 to 3.2)	1.2 (−2.6 to 5.0)

Abbreviation: GCS, Glasgow Coma Scale.

^a^ Scores range from 3 (lowest consciousness) to 15 (highest consciousness).

No increase in adverse events was observed beyond 30 minutes in the subset of patients for whom extended follow-up data were available (9 of 49 [18.4%] in the titrated IV morphine plus placebo group vs 5 of 40 [12.5%] in the titrated IV morphine plus acetaminophen group; *P* = .56). No patient experienced a serious adverse event requiring withdrawal from the study, nor did any require medical intervention to manage an adverse event.

## Discussion

In this randomized clinical trial of adults with acute pain seen in the ED, titrated IV morphine plus placebo did not demonstrate noninferiority compared with IV morphine plus acetaminophen for pain relief for either traumatic or nontraumatic pain. These findings render the primary outcome inconclusive and suggest that the potential benefit of adjunctive acetaminophen should not be dismissed.

While several postoperative trials and meta-analyses, including a Cochrane review,^29^ suggest that opioid efficacy increases when combined with paracetamol without increasing adverse effects, this benefit has not been consistently replicated in emergency care settings. Previous studies conducted in the ED have repeatedly failed to show a clinically meaningful improvement in pain scores with the addition of acetaminophen to opioid therapy.^7,8,9,10,11,12,13^ As a result, the prevailing view has been that acetaminophen offers limited or no added value in this setting. However, these trials were frequently constrained by methodologic issues—such as fixed opioid dosing, inconsistencies in opioid regimens, and heterogeneity in pain etiologies—that may compromise the robustness and applicability of their conclusions (eTable 1 in Supplement 2). Such limitations may have obscured a genuine benefit of acetaminophen in specific clinical contexts.

Our trial was designed to overcome these limitations using a rigorous noninferiority approach, stratified randomization based on pain type, weight-based IV morphine titration, and prespecified subgroups for traumatic and nontraumatic pain. These methodologic enhancements allowed for a more refined assessment of acetaminophen’s role in multimodal analgesia. The titration of IV morphine after the initial bolus likely enhanced the generalizability of our trial by reflecting actual ED practice, in which opioids are adjusted according to patient response. However, this approach may also have reduced the capacity to detect incremental benefit from acetaminophen, as titration can equalize analgesic response across treatment groups. Consequently, our results may underestimate the potential additive effect of acetaminophen when administered with a fixed or single morphine dose.

Notably, among patients with nontraumatic pain, the 95% CIs for the between-group differences in mean NRS pain score reduction fell entirely above 0, suggesting that IV morphine plus placebo may be inferior to combination therapy in this subgroup. This finding challenges the prevailing conclusion that acetaminophen is ineffective and suggests that prior trials may have failed to detect a meaningful benefit due to design limitations. Consistent with findings from oral analgesic studies, including a Cochrane review demonstrating improved pain relief when acetaminophen is combined with other oral analgesics, our results suggest that the adjunctive effect of acetaminophen may vary depending on route of administration, pain type, and dosing strategy.^30^ While our results do not establish superiority of the combination approach, they cast doubt on the assumption of equivalence and support reconsideration of acetaminophen as a useful adjunct to morphine, particularly when analgesia is tailored to the individual patient. This perspective is further supported by recent reviews such as that by Rech et al,^31^ which acknowledged that despite limited ED-based evidence of IV acetaminophen’s superiority vs placebo, its role in multimodal regimens—particularly for patients unable to tolerate oral analgesics—remains relevant and that it is potentially underused.

Nontraumatic pain—most notably neuropathic pain resulting from nerve injury and nociplastic pain driven by central sensitization—encompasses a broad range of mechanisms and is often less responsive to conventional opioid-based regimens.^32,33,34^ In such cases, multimodal analgesia strategies that incorporate IV acetaminophen may improve pain control while potentially reducing overall opioid requirements.^35,36^ These findings warrant further investigation into individualized treatment approaches for acute pain in the ED, particularly in populations with complex pain pathophysiology.

### Limitations

This study has several limitations. First, recruitment was prolonged by the COVID-19 pandemic, which caused temporary enrollment suspensions and delayed site reactivation; pandemic-related changes in staffing, patient flow, and care delivery may have introduced unmeasured confounding. These disruptions also limited enrollment within each prespecified pain stratum, reducing the planned precision, particularly for traumatic pain. Second, follow-up was limited to 60 minutes, reflecting early ED care but not capturing delayed effects, opioid use beyond the initial period, or later adverse events; longer follow-up with repeated dosing could better characterize longer-term outcomes. Third, because the analysis plan did not prespecify formal inferiority testing if noninferiority was not demonstrated, interpretation is limited for nontraumatic pain, where the 95% CI excluded 0, and warrants further study. Fourth, we did not assess other patient-centered outcomes (eg, time to meaningful pain relief, satisfaction, function, or ED length of stay). Fifth, despite stratification and standardized morphine titration, heterogeneity within pain strata and unmeasured history of chronic pain among participants may have influenced analgesic responsiveness.

## Conclusions

In this randomized clinical trial of adults with acute traumatic or nontraumatic pain seen in the ED, morphine plus placebo did not meet the criterion for noninferiority compared with morphine plus acetaminophen for pain relief during the initial 60 minutes of management. The findings suggest acetaminophen may have potential benefit as an adjunct to morphine in individualized treatment approaches for acute pain in the ED.

## Notes

Supplement 1.
Trial Protocol

Supplement 2. **eMethods.** Consent and Case Report Form Completion **eTable 1.** Summary of Prior Randomized Trials on Paracetamol or Comparator With Morphine in Acute Pain **eTable 2.** Number of Inclusions for Each Investigative Center **eTable 3.** Changes in Vital Signs Along Follow-Up in Patients With Acute Traumatic Pain, on Enrollment and at 10-Minute or 15-Minute Intervals Thereafter Until 60 Minutes After the Administration of the Study Medication **eTable 4.** Changes in Vital Signs in Patients With Acute Nontraumatic Pain, on Enrollment and at 10-Minute or 15-Minute Intervals Thereafter Until 60 Minutes After Administration of the Study Medication **eFigure.** Pain Severity Reduction by Trial Group on Enrollment and at 10-Minute or 15-Minute Intervals Thereafter Until 60 Minutes After the Administration of the Study Medication

Supplement 3.
Data Sharing Statement
