# Supplementary material for: Morphine Plus Placebo vs Morphine Plus Acetaminophen for Acute Pain in the Emergency Department: A Randomized Clinical Trial
Source: JAMA Netw Open. 2026 Feb 24;9(2):e2560250. doi: 10.1001/jamanetworkopen.2025.60250 (PMC12933283; doi:10.1001/jamanetworkopen.2025.60250)
Supplement: Supplement 1. — Trial Protocol [file jamanetwopen-e2560250-s001.pdf]

Morphine Plus Placebo vs Morphine Plus Acetaminophen for Initial Acute Pain Management in Emergency Department Patients: A Randomized Clinical Trial

N° enregistrement : n° 2019-002149-39
Réf. : RC19_0116

BIOMEDICAL RESEARCH PROTOCOL
Version N° 4 du 21/02/2024

which has received the favourable opinion of the CPP (Institutional Review Board), the date, and the authorization of the ANSM

This biomedical research project will be funded by funding source

Sponsor:

Nantes University Hospital
Medical Affairs and Research Department
5, allée de l'île Gloriette
44 093 Nantes cedex 01 (FRANCE)
Tel: 33 (0)2 53 48 28 35 70
Fax : 33 (0)2 53 48 28 36 71

Coordinating researcher:

Guillaume Cattin, emergency physician
Nantes University Hospital
02 53 48 20 38 76
emmanuel.montassier@chu-nantes.fr

Scientific director:

Emmanuel Montassier, emergency physician
Nantes University Hospital
02 53 48 20 38 76
emmanuel.montassier@chu-nantes.fr

Methodological support:

Christelle VOLTEAU
Direction de la Recherche et de l'Innovation
Plateforme Méthodologie et Biostatistique
5, allée de l'île Gloriette
44 093 Nantes cedex 01 (FRANCE)

Safety and Surveillance Unit for Clinical Research:

Dr Anne CHIFFOLEAU
Direction de la Recherche,
Département Promotion, Cellule Vigilances
CHU de Nantes,
5, allée de l'Ile Gloriette
44093 Nantes cedex 1
Tel : +33(0) 2 44 76 67 82 92
Fax : +33(0)2 53 48 28 36 93

LIST OF ABBREVIATIONS

ADR	Adverse Drug reaction
AE	Adverse Event
ANSM	Agence Nationale de Sécurité des Médicaments et des produits de santé
AMM	Autorisation de Mise sur le Marché
CPP	Comité de Protection des Personnes
CNIL	Commission Nationale de l'Informatique et des Libertés
CRA	Clinical Research Assistant
DSMB	Data Safety Monitoring Board
DSUR	Development Safety Update Report
e-CRF	Electronic Case Report Form
ED	Emergency Department
GCP	Good Clinical Practices
ICH	International Conference on Harmonisation
IP	Investigational Product
INSERM	Institut National de la Santé et de la Recherche Médicale
ITT	Intention to Treat
MR	Méthodologie de référence
REC	Research Ethics Committee
SAE	Serious Adverse Event
SmPC	Summary of Product Characteristics
SOP	Standard Operating Procedures

Table des matières

1. Rationale of the Study ***5***

1.1 Research Context **5**

1.2 Morphine as Standard Treatment **6**

1.3 Role of Paracetamol **6**

1.4 Unanswered Clinical Questions **6**

2. Study Feasibility *6*

3. Objectives and Endpoints *6*

3.1 Primary Objective 6

3.2 Primary Endpoint 7

3.3 Secondary Objectives 7

4. Study Design *7*

4.1 Treatment Groups 7

5. Study Population *7*

5.1 Inclusion Criteria 7

5.2 Exclusion Criteria 8

6. Treatments Used During the Study *8*

6.1 Administration 8

6.2 Blinding and Randomization 8

6.3 Prohibited Treatments 8

7. Study Flow and Timeline *9*

8. Data Management and Statistical Analysis *9*

8.1 Statistical Plan 9

9. Pharmacovigilance *9*

10. Regulatory and Ethical Considerations *14*

11. consent form *15*

1. Rationale of the Study

1.1 Research Context

Pain is often referred to as the fifth vital sign and is a common complaint among patients admitted to emergency departments. Approximately 78% of these patients experience pain, with traumatic pain accounting for 40% and abdominal or urological pain each contributing 13%. Despite its prevalence, pain is frequently undertreated in emergency settings, with studies highlighting substantial gaps in analgesic administration.

Various studies have shown that only a fraction of patients presenting with moderate to severe pain receive appropriate pain relief. In France, 30% of patients

discharged from emergency departments still experience unrelieved moderate to severe pain. This highlights the importance of improving pain management strategies as a public health priority.

1.2 Morphine as Standard Treatment

Morphine remains the primary opioid used to treat moderate to severe pain in emergency medicine. It is the most commonly administered parenteral opioid in U.S. emergency departments, and French expert recommendations advocate its use either alone or as part of a multimodal analgesic strategy.

1.3 Role of Paracetamol

Paracetamol is widely prescribed in emergency settings and recognized for its role in treating mild to moderate pain. However, while it is often used in combination with opioids like morphine, this combination lacks robust evidence of superiority over morphine alone in emergency settings. Moreover, paracetamol carries risks of hepatotoxicity, particularly at higher doses or in vulnerable patients.

1.4 Unanswered Clinical Questions

While multimodal analgesia is commonly promoted, especially in postoperative care, the effectiveness of combining paracetamol with morphine for acute pain in emergency settings remains unclear. Prior studies in this area often suffer from significant methodological limitations or target different patient populations. There is currently no high-quality evidence supporting routine paracetamol use with morphine for emergency analgesia.

2. Study Feasibility

Acute pain is a common reason for emergency department visits, with an estimated prevalence of 75%. At CHU Nantes, 829 patients were treated for acute pain in 2017, though this figure is likely underestimated due to data system changes.

The study involves eight emergency departments across France, ensuring a representative sample and robust inclusion potential. All participating centers are experienced in clinical research, including multicenter trials. The lead team has a strong background in clinical studies, as evidenced by their previous publications.

3. Objectives and Endpoints

3.1 Primary Objective

To assess the non-inferiority of intravenous morphine alone compared to the combination of intravenous morphine and paracetamol, 30 minutes after

administration, in patients presenting to the emergency department with moderate to severe pain (Numerical Verbal Scale [NVS] score ≥ 5), both traumatic and non-traumatic.

3.2 Primary Endpoint

The primary endpoint is the change in pain intensity measured by the NVS between baseline and 30 minutes after the first administration of the study treatment.

3.3 Secondary Objectives

Secondary objectives include evaluating pain relief at multiple time points (10, 20, 30, 45, 60 minutes), comparing total morphine consumption, success rate (NVS ≤ 3 at 30 minutes), adverse event rates, need for rescue analgesia, and changes in vital signs. These parameters aim to capture the overall analgesic efficacy and safety of the two treatment strategies.

4. Study Design

This is a Phase IV, prospective, multicenter, randomized, double-blind, placebo-controlled, non-inferiority clinical trial. It includes two parallel arms, stratified by pain type (traumatic vs. non-traumatic).

Patients will receive either intravenous morphine and a placebo or intravenous morphine and paracetamol. The total duration of the study is 61 months, with each patient followed until the end of emergency department care.

4.1 Treatment Groups

Group A: Morphine IV + Placebo
Group B: Morphine IV + Paracetamol IV

All patients will be assessed at 10, 20, 30, 45, and 60 minutes post-injection using NVS and vital sign monitoring. The final study visit occurs at discharge from the emergency department.

5. Study Population

A total of 572 patients, aged 18 or older, with moderate to severe acute pain (NVS ≥ 5) and clinical stability will be enrolled. Patients must be conscious and able to verbally assess their pain.

5.1 Inclusion Criteria

- Age ≥ 18 years
- NVS score ≥ 5

- Conscious (Glasgow score = 15)
- Clinically stable
- Able to verbalize pain level
- Covered by national health insurance

5.2 Exclusion Criteria

- Inability to consent
- Pregnant or breastfeeding women
- Body weight < 50 kg
- Severe cardiac, respiratory, hepatic, or renal conditions
- Prior analgesic use within 8 hours
- Known allergy to morphine or paracetamol
- Chronic pain under treatment

6. Treatments Used During the Study

Patients meeting all eligibility criteria will be randomized to receive either intravenous morphine plus a placebo or intravenous morphine plus paracetamol.

6.1 Administration

Morphine: 0.1 mg/kg IV initial bolus (maximum dose, 10 mg), titrated every 10 minutes with 0.05 mg/kg boluses (maximum, 5 mg per bolus), aiming for pain score ≤ 3 or until adverse effects occur.
Paracetamol: 1 gram IV, no dose adjustment.
Placebo: 100 mL sodium chloride 0.9%, visually indistinguishable from paracetamol.

6.2 Blinding and Randomization

Randomization is done 1:1 via Clinsight, stratified by pain type. Blinding is ensured by masking paracetamol and placebo vials. Morphine remains open-label due to safety requirements.

6.3 Prohibited Treatments

No additional morphine or paracetamol can be given within 30 minutes of study drug administration.
Naloxone and N-acetylcysteine can be administered in case of overdose.

7. Study Flow and Timeline

Patients will be evaluated at baseline and at 10, 20, 30, 45, and 60 minutes after study drug administration for pain level (NVS), vital signs, and adverse events. A final assessment will be done before discharge.

8. Data Management and Statistical Analysis

Data is collected using an electronic case report form (eCRF). Patient identification will use a coded number with minimal personal information.

8.1 Statistical Plan

The hypotheses for sample size calculations integrated the results of a large randomized clinical trial that compared the efficacy of 4 oral analgesics to treat acute pain in the emergency department. In this study, the largest difference in decline in the NRS pain score was 0.9. After assuming a noninferiority margin of 1 with a type I error of 5%/2 and type II error of 20%, we determined that 428 patients were needed (107 in each treatment group). To address missing pain assessments at 30 minutes, a worst-case imputation strategy was applied. For these patients, the smallest observed NRS change from baseline to 30 minutes among those with the same pain category and treatment group was imputed.**9. Pharmacovigilance**

All adverse events will be monitored, recorded in the eCRF, and classified by intensity. Serious adverse events (SAEs) must be reported to the sponsor without delay. A Data Safety Monitoring Board is not required.

8.2 Population definition

**Populations**	
Population	Definition
Intention-to-treat (ITT)	All randomized patients will be analyzed, including those for whom the ethical and administrative criteria have not been verified (for these patients, the data will be deleted, and all data used to calculate the primary endpoint will be imputed).
Modified Intention-to-treat (mITT)	The following are removed from the mITT population:<br>- Patients who withdrew consent to participate

	- Patient under guardianship<br>- Patient under 18 years old
Per Protocol (PP)	Removed from the PP population:<br><br>- Patients excluded from the mITT analysis<br>- Patients not meeting major inclusion/non-inclusion criteria<br>- Patients receiving rescue analgesia before T30<br>- Patients for whom the primary endpoint was not available<br>- Patients who did not receive the treatment assigned to them by randomization

8.3 Descriptive analyses

Characteristics of patients in each group will be summarized in a descriptive table. Descriptive statistical analysis will include for each quantitative variable: the mean, the standard deviation, the minimums and maximums, as well as the median and the quartiles. The qualitative variables will be expressed as frequencies and proportions. The standardized difference between the two groups will also be calculated for each variable and presented in this same table.

8.4 Management of missing data

Prior to the analyses, a completion of the missing data of primary outcome will be carried out, if necessary. Imputations will be made for the primary outcome by the average of the values of the patients in the same group. No imputations will be made for secondary endpoints. To address missing pain assessments at 30 minutes, a worst-case imputation strategy was applied. For these patients, the smallest observed NRS change from baseline to 30 minutes among those with the same pain category and treatment group was imputed.

8.5 Statistical analyses

Analyses of the primary outcome and the secondary outcomes will be presented in a summary table. Qualitative variables will be presented as frequencies and proportions. Quantitative variables will be presented as mean and standard deviation. The ordinal

variables will be presented as median and quartiles. Analyses will be done using SAS software version 9.4.

8.6 Analysis of primary outcome

The non-inferiority between the difference in mean change in verbal rating scale pain scores among patients receiving titrated IV morphine alone or titrated IV morphine plus acetaminophen, measured from the time before administration of the study medication to 30 minutes later will be tested using the confidence interval method. The bilateral confidence intervals at 95% of the difference titrated IV morphine alone minus titrated IV morphine alone with intravenous acetaminophen will be calculated using linear regression adjusted on baseline pain. The lower bounds of these confidence intervals must be superior to the non-inferiority limit defined at -1. The analysis will be performed per protocol and in Intent-to treat as recommended for non-inferiority trials. To address missing pain assessments at 30 minutes, a worst-case imputation strategy was applied. For these patients, the smallest observed NRS change from baseline to 30 minutes among those with the same pain category and treatment group was imputed.

8.7 Analyses of secondary outcomes

All secondary outcomes will be analyzed in traumatic and non-traumatic groups. All statistical tests will be two-sided at the 5% level of significance, except the noninferiority analysis of the evolution in pain change from baseline that will be analyzed as primary outcome.

8.7.1 NVS pain scores change from the time before administration of the study medication to 10, 20, 30, 45 and 60 minutes

Mixed linear regression models will be used to demonstrate the noninferiority of the group Placebo compared to the group IV acetaminophen, in order to taking into account repeated measures in the time. An adjustment on baseline pain value will be done. The 95% confidence interval of the difference between the 2 groups will be estimated: the noninferiority will be demonstrated if the lower bound of the confidence interval is superior to -1.

8.7.2 Cumulative dose of morphine at 60 minutes

The comparison between the two arms will be done with Student t tests.

8.7.3 Successful analgesia at 30 minutes

The comparison between the two arms will done with Chi-square tests.

8.7.4 Number of prescriptions of rescue analgesic molecules at 30 min

The comparison between the two arms will done with Chi-square tests.

8.7.5 Adverse events

The proportions of adverse events (serious and non-severe) will be described and compare with Chi-square and Fisher tests.

8.8 Subgroup analyses

We planned to analyze the primary outcome separately on the 2 following subgroups: traumatic pain and non-traumatic pain. The sample size was calculated to have sufficient power to analyze them separately.

8.9 Interim analysis

No interim analysis is planned.

8.10 Tables templates

The table templates are shown below.

Table 1. Demographic data and injury characteristics of patients.

Characteristics	All patients (n=)	Acetaminophen Group (n=)	Placebo Group (n=)
**Female, No. (%)**			
**Age, y**<br>Median (IQR)<br>Minimum, maximum			

Table 2. Secondary end points by Study Group

	Patient group[a]			
	**Paracetamol**	**Placebo**	**Risk difference**	***P* value**
Total amount of morphine at 30 minutes (mg), Median (IQR), non-traumatic pain		12 (8.8-15)		

Table 3. Frequency of adverse effects observed, by study group.

	Acetaminophen Group (n=)	Placebo Group (n=)	Risk Difference (acetaminophen–Placebo Group)

**Adverse Effect**	Frequency	Risk, %	95% CI	Frequency	Risk, %	95% CI	Risk Difference, %	95% CI
Nausea								

Supplemental Table. Vital sign changes during out-of-hospital management for pain by study group.

**Parameter**	**Acetaminophen Group (n=)**	**Placebo Group (n=)**	**Risk Difference (Acetaminophen-Placebo Group)**	**p-value**
**Pulse rate, mean beats/min**<br>T_0<br>T_{30}<br>Mean change*<br>95% CI				

10. Regulatory and Ethical Considerations

The protocol will be submitted for approval to the relevant Ethics Committee and ANSM. Informed consent must be obtained in writing. Patient confidentiality will be maintained in compliance with CNIL regulations.

11. consent form

CENTRE HOSPITALIER UNIVERSITAIRE DE NANTES<br>Version n° 1.1<br>Date : 17/09/2019	**ANNEXE 4**<br>**Certificate of consent to research**<br>**« ADAMOPA trial: Intravenous Acetaminophen with Morphine versus Intravenous Morphine Alone for Acute Pain in the Emergency Room: a Multicenter, Randomized, Placebo-controlled, Double-blinded Study»**<br>**Responsible party*: CHU de Nantes***<br>***N° EudracT :* 2019-002149-39.**

I, the undersigned
Me, M. (delete as appropriate) (first name, LAST NAME)……………………………………………………………………
……,

Date of Birth: …../……/……..
freely and voluntarily accepts to participate in the research referenced above, coordinated by the Doctor X, and organized by the Nantes University Hospital, promoter of research.

Being heard that :

- he investigator who informed me and answered all of my questions clearly, told me that my participation is free and that I can withdraw from the research at any time.
- I certify that I am not subject to protective measures (guardianship, curatorship, safeguard of justice), in addition I confirm that I am affiliated or benefit from a social security scheme.
- I was previously given an information note on this research specifying its purpose, methodology, expected benefits and foreseeable risks.
- I may have information from the investigator, during or after the research, of the information he has regarding my health.
- If the number of people foreseen in the study has been reached, I could ultimately not be included in the study even though I signed a consent. In this specific case, I will no longer be followed within the framework of the study and the data concerning me as well as the blood samples if necessary will be destroyed. This will in no case prejudice the quality of my care.
- I am fully aware that I can withdraw my consent to my participation in this research at any time, whatever my reasons and without bearing any responsibility, but I undertake in this case to inform the investigator . The fact of no longer participating in this research will not affect my relations with this investigator, nor the quality of the care that will be given to me.
- If I wish, the support person I have designated can assist me in my efforts and can attend medical interviews to help me in my decisions.

• I accept that my attending physician will be informed of my participation in the research:

☐ Yes, I accept ☐ No i refuse

- I may at any time request additional information from the investigator.
- If I wish, at the end, I will be informed by the investigator of the overall results of this research.
- My consent in no way relieves the investigator and the promoter of all their responsibilities and I retain all of my rights guaranteed by law.
- I accept that the data recorded during this research may be subject to computerized processing by the promoter or on his behalf, and I certify that I have been informed of all my rights concerning my personal data according to the terms and conditions described in the information note in force, which was sent to me for this protocol.

**PERSON INCLUDED IN THE RESEARCH**	
**Dated :**<br>**…… / ….. / ………..**	**Signature :**

**INVESTIGATOR: I certify that I have fully explained to the signatory the purpose, the procedures and the potential risks of the research**		
**Dated :**<br>**…… / ….. / ………..**	**NAME:**	**Signature :**

This document must be produced in 3 original copies: the first must be kept by the investigator and the second is given to the person giving his consent. In the event of a duplicate, the original is kept by the investigator and a copy is given to the person who gave his consent. In the event of a triplicate, the promoter will collect one of the duplicate consents in sealed envelopes throughout the study.
