# Supplementary material for: Morphine Plus Placebo vs Morphine Plus Acetaminophen for Acute Pain in the Emergency Department: A Randomized Clinical Trial
Source: JAMA Netw Open. 2026 Feb 24;9(2):e2560250. doi: 10.1001/jamanetworkopen.2025.60250 (PMC12933283; doi:10.1001/jamanetworkopen.2025.60250)
Supplement: Supplement 2. — eMethods. Consent and Case Report Form Completion eTable 1. Summary of Prior Randomized Trials on Paracetamol or Comparator With Morphine in Acute Pain eTable 2. Number of Inclusions for Each Investigative Center eTable 3. Changes in Vital Signs Along Follow-Up in Patients With Acute Traumatic Pain, on Enrollment and at 10-Minute or 15-Minute Intervals Thereafter Until 60 Minutes After the Administration of the Study Medication eTable 4. Changes in Vital Signs in Patients With Acute Nontraumatic Pain, on Enrollment and at 10-Minute or 15-Minute Intervals Thereafter Until 60 Minutes After Administration of the Study Medication eFigure. Pain Severity Reduction by Trial Group on Enrollment and at 10-Minute or 15-Minute Intervals Thereafter Until 60 Minutes After the Administration of the Study Medication [file jamanetwopen-e2560250-s002.pdf]

Supplementary Online Content

Cattin G, Viglino D, Segard J, et al. Morphine plus placebo vs morphine plus acetaminophen for acute pain in the emergency department: a randomized clinical trial. *JAMA Netw Open*. 2026;9(2):e2560250. doi:10.1001/jamanetworkopen.2025.60250

eMethods. Consent and Case Report Form Completion

eTable 1. Summary of Prior Randomized Trials on Paracetamol or Comparator With Morphine in Acute Pain

eTable 2. Number of Inclusions for Each Investigative Center

eTable 3. Changes in Vital Signs Along Follow-Up in Patients With Acute Traumatic Pain, on Enrollment and at 10-Minute or 15-Minute Intervals Thereafter Until 60 Minutes After Administration of the Study Medication

eTable 4. Changes in Vital Signs in Patients With Acute Nontraumatic Pain, on Enrollment and at 10-Minute or 15-Minute Intervals Thereafter Until 60 Minutes After the Administration of the Study Medication

eFigure. Pain Severity Reduction by Trial Group on Enrollment and at 10-Minute or 15-Minute Intervals Thereafter Until 60 Minutes After the Administration of the Study Medication

This supplementary material has been provided by the authors to give readers additional information about their work.

© 2026 Cattin G et al. *JAMA Network Open*.

eMethods. Consent and Case Report Form Completion

Process for obtaining consent

The treating attending confirmed that the patient has met enrollment criteria and assess whether the patient has capacity to provide informed consent. All participants provided written informed consent.

Onsite case report form completion

Each physician completed an electronic case report form (CRF). Later, in order to ensure the quality and completeness of the study data, a clinical research associate at each center verified the CRF data from the source medical file on-site and recorded the data to a centralized database. All 11 participating sites completed identical CRF for each patient enrolled in the study.

© 2026 Cattin G et al. *JAMA Network Open*.

eTable 1. Summary of Prior Randomized Trials on Paracetamol or Comparator With Morphine in Acute Pain.

First author (Year, location)	Sample size (Randomized)	Population	Intervention Comparator	Sample size calculation	Primary outcome	Main findings	Limitations
Farnia et al. (2016, Iran)	87	biliary pain	- group A: 0.05 mg/kg morphine + 1g paracetamol IV<br>- group B: 0.1 mg/kg morphine IV	A 15 mm difference in the mean VAS pain score, was considered clinically significant	VAS at 30 min	No significant difference: 1.66±1.59 in group A vs. 2.14±1.79 in group B; mean difference –0.48, and 95%CI –1.20 to 0.24 (P=0.19)	- inclusion criteria : VAS≥3 (patients not requiring morphine usually if VAS<5)<br>- bicentric study<br>- Underpowered,<br>- limited to 30min outcomes,<br>- sample size not adequate to detect adverse events<br>- different dose of morphine in the 2 groups of treatment (paracetamol and low-dose morphine versus high-dose morphine).
Minotti et al. (2022, Switzerland)	202	ED patients >18 years with acute pain (VAS> 4)	- 0.1 mg/kg morphine + 1g paracetamol IV<br>- 0.1 mg/kg morphine + placebo IV	Added effect of acetaminophen to reduce the mean morphine dose by 20% was considered clinically significant	mean morphine dose for pain relief	No significant difference: 0.15 ± 0.07 mg/kg or 12.0 ± 5.8 mg in the acetaminophen group, and 0.15 ± 0.07 mg/kg or 13.0 ± 6.2 mg in the placebo group (p=0.2).	- study designed to demonstrate superiority but failed to demonstrate superiority : underpowered for non-inériority or equivalence<br>- <5% of randomized patients among screened patients<br>- Heterogeneous population: patients presenting with pain of different origin, location, and quality, underpowered for subgroup analysis<br>- bicentric study
Zare et al. (2014, Iran)	90	Acute bone fracture pain	- intravenous morphine sulfate<br>- oral oxycodone plus intravenous acetaminophen	No detail on the sample size calculation	pain intensity at 30 and 60 min	No significant difference in pain reduction between groups	- Different route of opioid administration (oral vs IV)<br>- limited to bone fractures<br>- limited to patients having 15–60 years old<br>- underpowered<br>- limited external validation: excluded patients with chronic respiratory, cardiac and renal failure<br>- Fixed dosage of medications: no titration of morphine
Blok et al. (2021, USA)	116	patients aged 18 years and older and	- IV morphine + IV paracetamol (1 g)	difference of 2 MEU between the two groups	opioid requirement, measured in	Opioid consumption	- Single-center,<br>- Heterogeneous population: underpowered for subgroup analysis

© 2026 Cattin G et al. *JAMA Network Open*.

		treated with opioids (level one trauma center)	- IV morphine + placebo		Morphine Equivalent Units (MEU) during Emergency Department stay.	in the acute phase was not significantly different (p=0.53) between patients receiving (10.0 MEU (IQR 7.5; 15.0)) and those not receiving acetaminophen: 10.0MEU (IQR 7.1; 15.0).	- prospective cohort study: no randomization of the included patients
Bijur et al. (2020, USA)	162	patients aged 21 to 64 years with acute pain (onset within 7 days) that was of sufficient severity to warrant use of intravenous opioids	- 1 mg of IV Hydromorphone + 1 g of IV acetaminophen<br>- 1 mg of IV Hydromorphone + placebo	difference of at least 1.3 NRS units in the improvement in NRS pain score between the two study groups	between group difference in improvement of NRS pain scores at 60 minutes	difference of 0.8 NRS units (95% confidence interval [CI] = -0.01 to 1.8)	- bicentric,<br>- 80% of included patients with Abdomen/flank pain: preponderance of abdominal pain in this study may limit generalization to other types of pain<br>- study designed to demonstrate superiority but failed to demonstrate superiority : underpowered for non-infériority or equivalence<br>- no specific cut-off to start opioids treatment : substantial variation in pain treatment practices, this group of patients is likely to be quite heterogeneous and skewed toward patients with more severe pain<br>- demographic composition : predominantly female and Latina, may also limit extrapolation to other populations<br>- Fixed dosage of medications : Receipt of additional analgesics by patients with inadequate pain control could bias the measure of change in pain, as pain ascribed to the study medication could be due to receipt of rescue medication

© 2026 Cattin G et al. *JAMA Network Open*.

Chang et al. (2019, USA)	162	adults aged 65 years and older with acute severe pain (onset within 7 days) that was of sufficient severity to warrant use of intravenous opioids	- 0.5 mg of IV hydromorphone and 1 g of IV Acetaminophen<br>- 0.5 mg of IV hydromorphone and 100 mL of normal saline placebo	difference of at least 1.3 NRS units in the improvement in NRS pain score between the two study groups	between group difference in improvement of NRS pain scores at 60 minutes	a difference of 0.6 NRS units (95% confidence interval [CI] = -0.4 to 1.5).	- bicentric,<br>- no detail on nature of pain : possible heterogeneous population<br>- study designed to demonstrate superiority but failed to demonstrate superiority : underpowered for non-infériority or equivalence<br>- Fixed dosage of medications : 29% of patients in each group requested additional analgesia at 60 minutes. Receipt of additional analgesics by patients with inadequate pain control could bias the measure of change in pain, as pain ascribed to the study medication could be due to receipt of rescue medication<br>- no specific cut-off to start opioids treatment : substantial variation in pain treatment practices, this group of patients is likely to be quite heterogeneous and skewed toward patients with more severe pain

Abbreviations: VAS : visual analogue pain score; ED: Emergency Department; MEU : Morphine Equivalent Units; NRS : numerical rating scale

© 2026 Cattin G et al. *JAMA Network Open*.

eTable 2. Number of Inclusions for Each Investigative Center

Center	No. of inclusions (N = 430)
CHU Nantes, n (%)	201 (46.7)
CH La Roche sur Yon, n (%)	4 (1)
CHU Angers, n (%)	9 (2)
CHU Bordeaux, n (%)	11 (2.6)
CHU Grenoble, n (%)	89 (20.7)
CHU Nancy, n (%)	2 (0.5)
CHU Pitié Salpetrière, n (%)	37 (8.6)
CHU Lariboisière, n (%)	41 (9.5)
CH Saint Nazaire, n (%)	23 (5.4)
CHU Rouen, n (%)	6 (1.4)
CH Lorient, n (%)	7 (1.6)

© 2026 Cattin G et al. *JAMA Network Open*.

eTable 3. Changes in Vital Signs Along Follow-Up in Patients With Acute Traumatic Pain, on Enrollment and at 10-Minute or 15-Minute Intervals Thereafter Until 60 Minutes After Administration of the Study Medication.

	**Patient group**			
**Parameter**	**Morphine plus acetaminophen (n=88)**	**Morphine plus placebo (n=93)**	**Adjusted difference mean**	***P* value**
**Pulse rate, mean (SD), beats/min**				
T0	80.1 (12.7)	76.5 (14.9)		
T10	77.1 (11.6)	75.6 (12.8)		
Mean change (95% CI)	2.5 (0.8 to 4.1)	1.6 (0.0 to 3.3)	-0.8 (-3.2 to 1.5)	0.47
T20	77.1 (11.3)	74.0 (12.8)		
Mean change (95% CI)	2.4 (0.7 to 4.1)	2.6 (1.0 to 4.3)	0.2 (-2.2 to 2.6)	0.85
T30	76.2 (10.7)	75.0 (14.5)		
Mean change (95% CI)	3.1 (1.4 to 4.9)	2.2 (0.5 to 3.9)	-0.9 (-3.4 to 1.5)	0.46
T45	74.8 (11.7)	73.9 (16.2)		
Mean change (95% CI)	4.3 (2.2 to 6.4)	3.1 (1.1 to 5.2)	-1.2 (-4.1 to 1.8)	0.42
T60	74.5 (12.2)	74.1 (15.6)		
Mean change (95% CI)	4.8 (2.6 to 7.0)	2.5 (0.4 to 4.6)	-2.3 (-5.4 to 0.7)	0.13
**Respiratory rate, mean (SD), breaths/min**				
T0	18.2 (4.1)	19.1 (4.2)		
T10	17.1 (3.6)	17.5 (3.9)		
Mean change (95% CI)	1.2 (0.5 to 2.0)	1.4 (0.6 to 2.2)	0.1 (-1.0 to 1.2)	0.80
T20	16.8 (3.9)	17.0 (3.8)		
Mean change (95% CI)	1.5 (0.7 to 2.3)	1.5 (0.7 to 2.3)	0.0 (-1.1 to 1.1)	0.99

© 2026 Cattin G et al. *JAMA Network Open*.

T30	16.4 (3.4)	17.6 (3.8)		
Mean change (95% CI)	2.0 (1.2 to 2.7)	1.3 (0.5 to 2.1)	-0.7 (-1.8 to 0.4)	0.22
T45	16.7 (3.6)	16.9 (3.7)		
Mean change (95% CI)	1.8 (1.0 to 2.5)	2.0 (1.2 to 2.7)	0.2 (-0.9 to 1.3)	0.75
T60	16.3 (2.8)	16.9 (3.8)		
Mean change (95% CI)	2.1 (1.5 to 2.8)	1.9 (1.2 to 2.6)	-0.2 (-1.2 to 0.7)	0.64
**Systolic blood pressure, mean (SD), mm Hg**				
T0	139.6 (22.9)	137.3 (22.6)		
T10	132.9 (22.1)	132.3 (20.3)		
Mean change (95% CI)	6.3 (2.9 to 9.6)	5.4 (2.0 to 8.7)	-0.9 (-5.6 to 3.9)	0.71
T20	132.6 (22.3)	131.3 (19.3)		
Mean change (95% CI)	6.2 (2.7 to 9.7)	5.6 (2.2 to 9.0)	-0.6 (-5.5 to 4.3)	0.81
T30	131.3 (21.6)	132.9 (22.8)		
Mean change (95% CI)	7.7 (4.2 to 11.1)	5.0 (1.7 to 8.4)	-2.6 (-7.4 to 2.2)	0.28
T45	126.8 (19.8)	132.2 (23.0)		
Mean change (95% CI)	11.2 (7.6 to 14.9)	6.5 (2.9 to 10.0)	-4.7 (-9.8 to 0.3)	0.07
T60	126.8 (20.1)	132.4 (21.6)		
Mean change (95% CI)	11.9 (8.4 to 15.3)	5.7 (2.3 to 9.0)	-6.2 (-11.0 to -1.4)	0.01
**Diastolic blood pressure, mean (SD), mm Hg**				
T0	80.6+/-13.7	81.2+/-13.0		
T10	77.2+/-13.8	77.6+/-14.6		
Mean change (95% CI)	3.5 (1.0 to 6.0)	3.2 (0.7 to 5.7)	-0.2 (-3.8 to 3.3)	0.89
T20	76.0+/-13.0	76.3+/-15.6		
Mean change (95% CI)	4.3 (1.7 to 6.9)	4.3 (1.7 to 6.9)	-0.0 (-3.7 to 3.7)	0.99

© 2026 Cattin G et al. *JAMA Network Open*.

T30	74.5+/-13.9	77.4+/-14.7		
Mean change (95% CI)	5.8 (3.2 to 8.5)	3.9 (1.3 to 6.6)	-1.9 (-5.7 to 1.8)	0.32
T45	73.3+/-13.0	74.5+/-13.7		
Mean change (95% CI)	6.8 (4.3 to 9.3)	6.6 (4.2 to 9.0)	-0.2 (-3.7 to 3.3)	0.91
T60	71.8+/-12.9	74.7+/-14.7		
Mean change (95% CI)	8.6 (6.2 to 11.0)	6.1 (3.8 to 8.4)	-2.5 (-5.9 to 0.8)	0.14

© 2026 Cattin G et al. *JAMA Network Open*.

eTable 4. Changes in Vital Signs in Patients With Acute Nontraumatic Pain, on Enrollment and at 10-Minute or 15-Minute Intervals Thereafter Until 60 Minutes After Administration of the Study Medication.

	**Patient group**			
**Parameter**	**Morphine plus acetaminophen (n=123)**	**Morphine plus placebo (n=120)**	**Adjusted difference mean**	***P* value**
**Pulse rate, mean (SD), beats/min**				
T0	78.7+/-15.3	78.9+/-14.3		
T10	75.5+/-14.3	74.3+/-12.9		
Mean change (95% CI)	3.1 (1.5 to 4.8)	4.9 (3.2 to 6.5)	1.7 (-0.6 to 4.0)	0.14
T20	73.8+/-14.1	74.9+/-13.2		
Mean change (95% CI)	4.8 (3.1 to 6.6)	4.4 (2.6 to 6.2)	-0.5 (-3.0 to 2.1)	0.72
T30	72.5+/-13.5	74.9+/-12.6		
Mean change (95% CI)	5.7 (3.9 to 7.4)	4.3 (2.6 to 6.1)	-1.3 (-3.8 to 1.1)	0.29
T45	72.6+/-14.2	75.8+/-13.4		
Mean change (95% CI)	5.5 (3.5 to 7.5)	3.5 (1.4 to 5.5)	-2.0 (-4.9 to 0.8)	0.17
T60	71.5+/-12.7	74.4+/-12.8		
Mean change (95% CI)	6.4 (4.5 to 8.2)	4.7 (2.9 to 6.6)	-1.6 (-4.3 to 1.0)	0.23
**Respiratory rate, mean (SD), breaths/min**				
T0	18.9+/-4.8	19.9+/-5.0		
T10	17.5+/-4.8	18.2+/-4.4		
Mean change (95% CI)	1.9 (1.1 to 2.6)	1.4 (0.6 to 2.1)	-0.5 (-1.5 to 0.6)	0.36
T20	17.1+/-5.0	18.1+/-4.0		
Mean change (95% CI)	2.1 (1.4 to 2.8)	1.6 (0.9 to 2.3)	-0.5 (-1.5 to 0.5)	0.32

© 2026 Cattin G et al. *JAMA Network Open*.

T30	16.9+/-3.5	17.4+/-3.5		
Mean change (95% CI)	2.0 (1.3 to 2.7)	2.1 (1.4 to 2.8)	0.1 (-0.9 to 1.1)	0.83
T45	16.7+/-3.8	17.4+/-3.2		
Mean change (95% CI)	2.2(1.5 to 2.9)	2.1 (1.4 to 2.8)	-0.1 (-1.1 to 0.9)	0.83
T60	16.3+/-3.4	17.5+/-3.2		
Mean change (95% CI)	2.6 (1.9 to 3.3)	2.0 (1.3 to 2.6)	-0.7 (-1.6 to 0.3)	0.18
**Systolic blood pressure, mean (SD), mm Hg**				
T0	131.0+/-23.1	134.8+/-20.0		
T10	126.3+/-19.3	126.6+/-17.2		
Mean change (95% CI)	5.4 (3.1 to 7.7)	7.1 (4.7 to 9.4)	1.7 (-1.6 to 5.0)	0.31
T20	122.7+/-18.4	128.9+/-17.6		
Mean change (95% CI)	8.5 (6.0 to 11.0)	5.0 (2.4 to 7.5)	-3.6 (-7.1 to -0.0)	0.05
T30	122.1+/-18.4	127.0+/-17.5		
Mean change (95% CI)	9.1 (6.8 to 11.5)	6.7 (4.3 to 9.1)	-2.5 (-5.8 to 0.9)	0.15
T45	120.8+/-19.4	126.5+/-17.4		
Mean change (95% CI)	10.3 (7.7 to 12.9)	7.2 (4.6 to 9.9)	-3.1 (-6.8 to 0.6)	0.10
T60	120.1+/-20.1	127.0+/-16.0		
Mean change (95% CI)	11.0 (8.5 to 13.6)	6.9 (4.3 to 9.5)	-4.2 (-7.8 to -0.5)	0.03
**Diastolic blood pressure, mean (SD), mm Hg**				
T0	77.2+/-12.9	78.1+/-11.9		
T10	75.5+/-12.1	76.3+/-11.4		
Mean change (95% CI)	1.8 (0.2 to 3.5)	1.5 (-0.2 to 3.2)	-0.3 (-2.7 to 2.1)	0.80
T20	73.0+/-12.2	75.4+/-13.2		
Mean change (95% CI)	4.2 (2.3 to 6.1)	2.4 (0.6 to 4.3)	-1.7 (-4.4 to 0.9)	0.20

© 2026 Cattin G et al. *JAMA Network Open*.

T30	72.6+/-12.1	74.6+/-12.1		
Mean change (95% CI)	4.4 (2.7 to 6.1)	3.3 (1.6 to 5.0)	-1.1 (-3.6 to 1.3)	0.36
T45	70.7+/-10.7	74.8+/-13.2		
Mean change (95% CI)	6.4 (4.5 to 8.2)	3.3 (1.4 to 5.1)	-3.1 (-5.8 to -0.5)	0.02
T60	69.7+/-11.2	73.6+/-12.3		
Mean change (95% CI)	7.3 (5.5 to 9.1)	4.4 (2.6 to 6.1)	-2.9 (-5.5 to -0.4)	0.02

© 2026 Cattin G et al. *JAMA Network Open*.

eFigure. Pain Severity Reduction by Trial Group on Enrollment and at 10-Minute or 15-Minute Intervals Thereafter Until 60 Minutes After the Administration of the Study Medication. A) in the group of ED patients with acute traumatic pain, B) in the group of ED patients with acute non-traumatic pain.

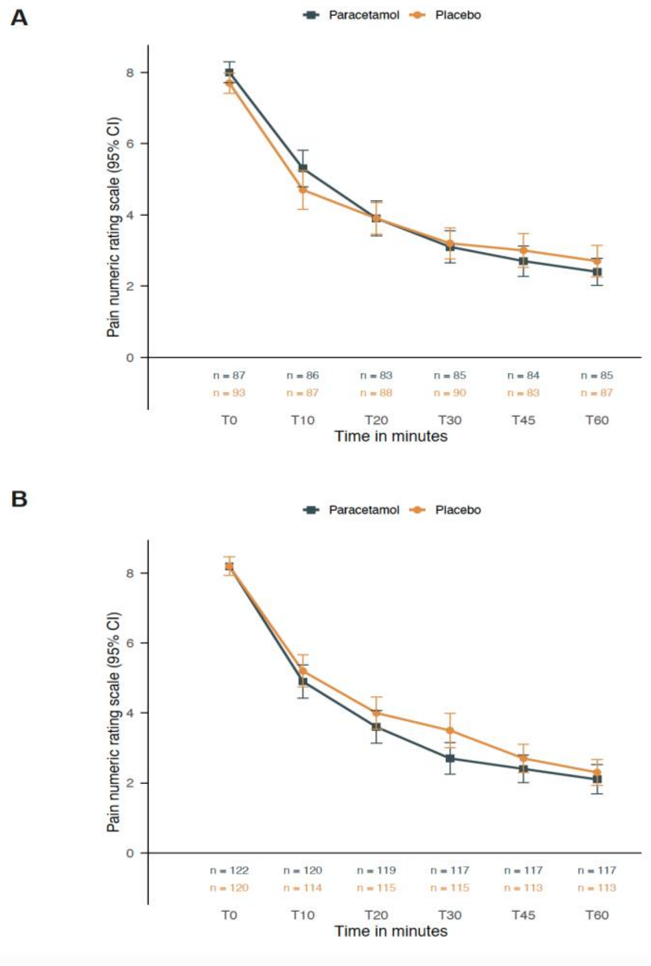

© 2026 Cattin G et al. *JAMA Network Open*.
