# Supplementary material for: Morphine Plus Placebo vs Morphine Plus Acetaminophen for Acute Pain in the Emergency Department: A Randomized Clinical Trial
Source: JAMA Netw Open. 2026 Feb 24;9(2):e2560250. doi: 10.1001/jamanetworkopen.2025.60250 (PMC12933283; doi:10.1001/jamanetworkopen.2025.60250)
Supplement: Supplement 3. — Data Sharing Statement [file jamanetwopen-e2560250-s003.pdf]

Data Sharing Statement

Cattin. Morphine Plus Placebo vs Morphine Plus Acetaminophen for Acute Pain in the Emergency Department. *JAMA Netw Open*. Published February 24, 2026. doi:10.1001/jamanetworkopen.2025.60250

Data
Additional Information: ClinicalTrials.gov Identifier: NCT04148495
Data available: Yes
Data types: Deidentified participant data
How to access data: Deidentified individual participant data that underlie the results reported in this article will be made available to researchers who provide a methodologically sound proposal and for the purpose of achieving the aims of the approved proposal. Data will be available upon reasonable request from the corresponding author (the principal investigator).
When available: With publication

Supporting Documents
Document types: Statistical/analytic code, Informed consent form
How to access documents: Supplement 3 Statistical analysis plan Supplement 2 Supplementary Online Content
When available: With publication

Additional Information
Who can access the data: Supplement 2 Supplementary Online Content
Types of analyses: for any purpose
Mechanisms of data availability: Deidentified individual participant data that underlie the results reported in this article will be made available to researchers who provide a methodologically sound proposal and for the purpose of achieving the aims of the approved proposal. Data will be available upon reasonable request from the corresponding author (the principal investigator).
